# Fractalkine suppression during hepatic encephalopathy promotes neuroinflammation in mice

**DOI:** 10.1186/s12974-016-0674-8

**Published:** 2016-08-26

**Authors:** Matthew McMillin, Stephanie Grant, Gabriel Frampton, Sarah Andry, Adam Brown, Sharon DeMorrow

**Affiliations:** 1 Department of Internal Medicine, Texas A&M Health Science Center, College of Medicine, Temple, TX USA; 2 Central Texas Veterans Healthcare System, 1901 S. 1st Street, Building 205, Temple, TX 76504 USA; 3 Department of Internal Medicine, Baylor Scott & White Health, 2401 S. 31st Street, Temple, TX 76508 USA

**Keywords:** Acute liver failure, Azoxymethane, CX3CL1, CX3CR1, CCL2

## Abstract

**Background:**

Acute liver failure is associated with numerous systemic consequences including neurological dysfunction, termed hepatic encephalopathy, which contributes to mortality and is a challenge to manage in the clinic. During hepatic encephalopathy, microglia activation and neuroinflammation occur due to dysregulated cell signaling and an increase of toxic metabolites in the brain. Fractalkine is a chemokine that is expressed primarily in neurons and through signaling with its receptor CX3CR1 on microglia, leads to microglia remaining in a quiescent state. Fractalkine is often suppressed during neuropathies that are characterized by neuroinflammation. However, the expression and subsequent role of fractalkine on microglia activation and the pathogenesis of hepatic encephalopathy due to acute liver failure is unknown.

**Methods:**

Hepatic encephalopathy was induced in mice via injection of azoxymethane (AOM) or saline for controls. Subsets of these mice were implanted with osmotic minipumps that infused soluble fractalkine or saline into the lateral ventricle of the brain. Neurological decline and the latency to coma were recorded in these mice, and brain, serum, and liver samples were collected. Neurons or microglia were isolated from whole brain samples using immunoprecipitation. Liver damage was assessed using hematoxylin and eosin staining and by measuring serum liver enzyme concentrations. Fractalkine and CX3CR1 expression were assessed by real-time PCR, and proinflammatory cytokine expression was assessed using ELISA assays.

**Results:**

Following AOM administration, fractalkine expression is suppressed in the cortex and in isolated neurons compared to vehicle-treated mice. CX3CR1 is suppressed in isolated microglia from AOM-treated mice. Soluble fractalkine infusion into the brain significantly reduced neurological decline in AOM-treated mice compared to saline-infused AOM-treated mice. Infusion of soluble fractalkine into AOM-treated mice reduced liver damage, lessened microglia activation, and suppressed expression of chemokine ligand 2, interleukin-6, and tumor necrosis factor alpha compared to saline-infused mice.

**Conclusions:**

These findings suggest that fractalkine-mediated signaling is suppressed in the brain following the development of hepatic encephalopathy. Supplementation of AOM-treated mice with soluble fractalkine led to improved outcomes, which identifies this pathway as a possible therapeutic target for the management of hepatic encephalopathy following acute liver injury.

## Background

Acute liver failure in Europe and the USA is caused primarily by acetaminophen-induced drug toxicity but can also be the result of viral hepatitis, toxin ingestion, non-acetaminophen-induced drug toxicity, and numerous other rare causative factors [1]. Following the development of acute liver failure, a variety of extrahepatic complications can develop including renal, respiratory, immunological, and neurological dysfunction. Of these complications, neurological dysfunction accounts for around 25 % of the mortality associated with this disease state and remains a significant challenge for clinical management of this disease [2]. These neurological complications are termed hepatic encephalopathy and have a variety of neuropsychiatric presentations from asymptomatic to severe cognitive decline and coma with implications on liver transplant priority, patient quality of life, and survival [3, 4].

A growing body of evidence implicates neuroinflammation and microglia activation involvement with the development of hepatic encephalopathy. Elevations of the proinflammatory cytokines interleukin (IL)-1β, IL-6, and tumor necrosis factor alpha (TNFα) are observed in both patients and in rodent models of acute liver failure [5, 6]. During hepatic encephalopathy, the use of anti-inflammatory therapeutics, such as minocycline, has been shown to reduce neuroinflammation and neurological dysfunction [7]. The production of pathogenic proinflammatory cytokines in the brain is a consequence of microglia activation, which can occur via a variety of signals including signal transduction by chemokines. We have previously shown that chemokine ligand 2 (CCL2) is one pathogenic chemotactic cytokine that is elevated in neurons during hepatic encephalopathy due to acute liver failure and contributes to both microglia activation and the elevation of IL-1β and IL-6 [8]. The receptors for CCL2 and other chemokines are primarily localized to microglia in the brain and therefore chemokine signaling is important in initiating the activation of microglia during states of neuroinflammation. Together, this implicates neuroinflammation and chemokine-signaling pathways as causative factors in the progression of hepatic encephalopathy.

Fractalkine, also known as CX3CL1, is the only member of the CX3C class of chemokines. In the brain, fractalkine is highly expressed in neurons but can be induced in astrocytes if they are treated with TNFα or interferon-γ [9, 10]. Fractalkine in its native form is a transmembrane protein that can be cleaved by cathepsin S or a disintegrin and metalloproteinase proteins (ADAMs) [11, 12]. Upon cleavage, fractalkine is released in its soluble form that contains the extracellular N-terminal chemokine domain. Soluble fractalkine can then bind its receptor, CX3CR1, which is a G-protein coupled receptor that is localized to microglia and is thought to keep microglia in a quiescent state [9, 13]. Fractalkine/CX3CR1 signaling has been demonstrated in numerous studies to dampen neuroinflammation, with fractalkine signaling being shown to reduce IL-1β secretion in lipopolysaccharide-treated microglia [14]. This has also been shown in vivo as rats with cerebral ischemia administered CX3CR1 siRNA were found to have increased microglia activation and proinflammatory cytokine expression compared to ischemic controls [15]. In addition to this, in a variety of inflammatory conditions there is a reciprocal relationship between fractalkine and CCL2, though whether these chemokines have a direct effect on each other or are inversely affected by an extraneous signal is unknown [16, 17].

At this time, there have been no studies investigating fractalkine in the context of neuroinflammation associated with hepatic encephalopathy. Therefore, the aims of this study were to assess the expression of fractalkine and its interaction with inflammatory signaling in a murine model of hepatic encephalopathy due to acute liver failure and to determine how fractalkine contributes to the neurological complications associated with this disease state.

## Methods

### Materials

Fractalkine antibodies were purchased from Abcam (Cambridge, MA). Antibodies against IBA1 were purchased from Wako Chemicals USA (Richmond, VA). NeuN antibodies were ordered from Millipore (Billerica, MA). Antibodies against CD11b and glutamate aspartate transporter (GLAST) were purchased from Miltenyi Biotec (San Diego, CA). Phosphorylated and total extracellular signal-regulated kinase 1/2 (pERK1/2 and tERK1/2) antibodies were purchased from Cell Signaling (Danvers, MA). Mouse soluble fractalkine was purchased from R&D Systems (Minneapolis, MN). Mouse fracktalkine, CCL2, IL-6, and TNFα ELISAs were purchased from R&D Systems. All real-time PCR (RT-PCR) primers were purchased from SABiosciences (Frederick, MD). Total bilirubin was assayed using an ELISA kit from Cusabio (Wuha, China). All other chemicals and reagents were purchased from Sigma-Aldrich (St. Louis, MO) unless otherwise noted and were of the highest grade available.

### Mouse model of hepatic encephalopathy

Male C57Bl/6 mice (20–25 g; Charles River Laboratories, Wilmington, MA) were used in all in vivo experiments using methodology described previously [8, 18, 19]. All animal experiments were performed following approval from Baylor Scott & White Health Institutional Animal Care and Use Committee (Temple, TX). Mice were provided free access to water and rodent chow and were housed in constant temperature, humidity, and 12-h light-dark cycling. Acute liver failure was induced via a single intraperitoneal injection of 100 mg/kg of azoxymethane (AOM) as previously described [18, 19]. In parallel, soluble fractalkine was administered to the brain via intracerebroventricular (ICV) infusion (200 ng/day) for 3 days prior to injection of AOM as previously described [18]. This dose was selected to maintain concentrations of fractalkine in the cerebrospinal fluid at concentrations that are at or above those reported by other researchers [20]. Care was taken to minimize the effects of anesthesia on hepatic metabolism in ICV mice by the following: (1) all mice were anesthetized with isoflurane to reach surgical plane with the subsequent surgeries taking approximately 30 min to complete per mouse and (2) AOM injection was delayed for 3 days after the ICV surgery to allow for residual anesthesia to be eliminated from the body. After AOM injection, mice were placed on heating pads adjusted to 37 °C and monitored for signs of neurological decline. To reduce the impact of hypoglycemia and dehydration, cage floors were supplied with hydrogel and rodent chow and after 12 h and every subsequent 4 h, mice were injected subcutaneously with 5 % dextrose in 250 μl of saline. If mice underwent a 20 % weight loss or greater, they were removed from the study.

At 12 h following injection (and every 2 h thereafter), body temperature, weight, and neurological assessments were measured. Neurological functioning was assessed by measuring the pinna reflex, corneal reflex, tail flexion reflex, escape response reflex, righting reflex, and ataxia which were scored on a scale of 0 (no reflex) to 2 (intact reflex). The neurological score at each time point was defined as the summation of these reflex scores. In addition, time to coma (defined as a loss of all reflexes) was recorded. Tissue was flash frozen and collected at coma for further analyses. Mice used for histochemical studies were transcardially perfused with PBS followed by 4 % paraformaldehyde. Whole brains were removed and placed into 4 % paraformaldehyde for 24 h, after which they were moved to a 30 % sucrose solution for cryoprotection. Brains were frozen and sectioned using a cryostat for immunofluorescence imaging.

### Cell isolation and flow cytometry

Fresh whole brains from adult C57Bl/6 mice were homogenized using an automated homogenizer from Miltenyi Biotec using solutions provided in the Neural Tissue Dissociation Kit (Miltenyi Biotec). Following dissociation into a single-cell suspension, cells were passed through LS columns (Miltenyi Biotec) containing beads coated with CD11b antibodies (to isolate microglia) or GLAST antibodies (to isolate astrocytes) localized to the columns. The remaining cells not bound to the columns were kept as the neuron-enriched fraction. LS columns were washed to remove the CD11b-bound cells or the GLAST-bound cells. Cells were subsequently spun down and homogenized for subsequent experiments.

### Liver histology and biochemistry

Paraffin-embedded livers were cut into 3-μm sections and mounted onto positively charged slides (VWR, Radnor, PA). Slides were deparaffinized and stained with Hematoxylin QS (Vector Laboratories, Burlingame, CA) for 1 min, followed by staining for 1 min with eosin Y (Amresco, Solon, OH), and rinsed in 95 % ethanol. The slides were then dipped into 100 % ethanol and subsequently through two xylene washes. Coverslips were mounted onto the slides using Vectamount mounting media (Vector Laboratories). The slides were viewed and imaged using an Olympus BX40 microscope with an Olympus DP25 imaging system (Olympus, Center Valley, PA).

Serum alanine aminotransferasase (ALT) and bilirubin were assessed using commercially available kits. Alanine aminotransferase measurement was performed using a fluorimetric activity assay. Total bilirubin was assayed using a total bilirubin ELISA. All assays and subsequent analyses were performed according to the instructions provided by the manufacturers.

### Real-time PCR

RNA was extracted from flash frozen tissue or isolated neural cells, and RT-PCR was performed as previously described using commercially available primers designed against mouse fractalkine, CX3CR1, and glyceraldehyde 3-phosphate dehydrogenase [21]. A ΔΔCT analysis was performed using vehicle-treated tissue or isolated cortical neurons/microglia as controls for subsequent experiments [22, 23]. Data for all experiments are expressed as mean relative mRNA levels ± SEM.

### Immunofluorescence

Free-floating immunostaining was performed on brain sections using anti-IBA1 immunoreactivity to detect morphology and relative staining of microglia. In addition, fractalkine and NeuN immunostaining were performed. Immunoreactivity was visualized using fluorescent secondary antibodies labeled with Dylight 488 or Cy3 and counterstained with ProLong© Gold Antifade Reagent containing 4′,6-diamidino-2-phenylindole (DAPI). Slides were viewed and imaged using a Leica Microsystems TCS SP5-X inverted confocal microscope (Buffalo Grove, IL). Quantification of IBA-1 field staining was performed by converting images to grayscale, inverting their color and quantifying the number of stained pixels with ImageJ software. The values for the vehicle group were normalized to 1 with this reference being used for all other quantifications of IBA1 field staining. Z-stack images were created by performing a 30-μm merge of images through the entire brain section using ImageJ software.

### Cytokine and chemokine ELISAs

Cortex tissue from all treatment groups was homogenized using a Miltenyi Biotec gentleMACS™ Dissociator, and total protein was quantified using a Pierce™ BCA Protein Assay kit (ThermoFisher Scientific, Waltham, MA). Capture antibodies against CCL2, IL-6, TNFα, or fractalkine were incubated overnight in 96-well plates. Each ELISA was performed according to the instructions provided from R&D Systems, and the total input protein for each sample was 50 μg. Absorbance was read using a SpectraMax® M5 plate reader from Molecular Devices (Sunnyvale, CA). Data are reported as CCL2, IL-6, TNFα, or fractalkine concentration per milligram of total lysate protein.

### Immunoblotting

Immunoblots were performed as previously described [8] with minor modifications. For western blots, 10 % sodium dodecyl sulfate-polyacrylamide gel electrophoresis gels were loaded with 20 μg of protein diluted in Laemmli buffer. Specific primary antibodies against pERK1/2 and tERK1/2 were used along with appropriate fluorescent secondary antibodies (LI-COR, Lincoln, NE). All imaging was performed using an Odyssey 9120 Infrared Imaging System (LI-COR). Data are expressed as fold change in fluorescent band intensity of pERK1/2 divided by tERK1/2. The values of the vehicle ICV saline group was used as a baseline and set to a relative protein expression value of 1. All band intensity quantifications were performed using ImageJ software (National Institutes of Health, Bethesda, MD). Data for all experiments were expressed as mean relative protein ± SEM (*n* = 4).

### Statistical analysis

All statistical analyses were performed using Graphpad Prism software (Graphpad Software, La Jolla, CA). Results were expressed as mean ± SEM. For data passing normality tests, significance was established using the Student’s *t* test when differences between two groups were analyzed, and analysis of variance when differences between three or more groups were compared, followed by the appropriate post hoc test. For neurological score analyses, two-way analysis of variance analysis and a subsequent Bonferroni post hoc test were performed. If tests for normality failed, two groups were compared with a Mann-Whitney *U* test or a Kruskal-Wallis ranked analysis when more than two groups were analyzed. Differences were considered significant when the *p* value was less than 0.05.

## Results

### Neuronal fractalkine was downregulated following AOM administration

**Fig. 1:**
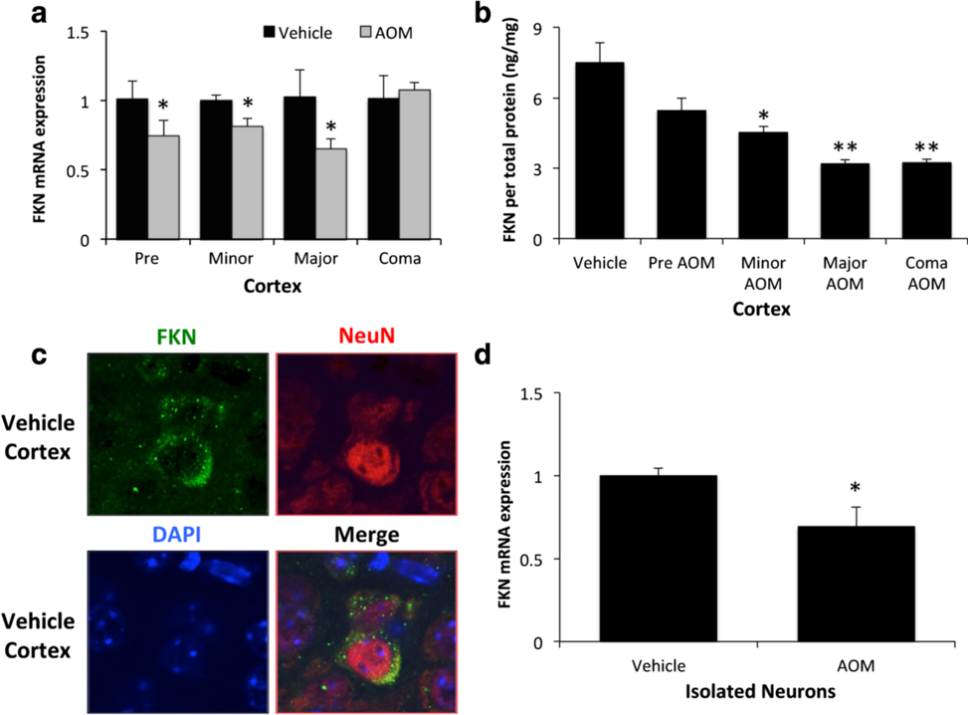
Neuronal fractalkine was downregulated following AOM administration. **a** Fractalkine mRNA expression in the cortex during the timecourse of AOM-induced HE with appropriate time-matched tissue analyses from vehicle-treated controls. **b** Fractalkine concentrations in cortex lysate normalized to protein concentrations of cortex lysates from vehicle and AOM-treated timecourse mice. **c** Immunofluorescence for fractalkine (*green*) and NeuN (*red*) with DAPI (*blue*) used as a nuclear counterstain in vehicle cortex brain tissue. **d** Fractalkine mRNA expression in neurons isolated from vehicle-treated and AOM-treated mice cortices. For mRNA expression and fractalkine ELISA analyses, *p < 0.05 compared to vehicle-treated mice or neurons from vehicle-treated mice, **p < 0.01 compared to vehicle-treated mice. *n* = 3 for fractalkine mRNA and ELISA analyses

C57Bl/6 mice were administered the hepatotoxin AOM to induce acute liver failure and the development of hepatic encephalopathy. The mouse AOM model of hepatic encephalopathy is characterized by a prodromal phase that transitions into mild cognitive deficits and quickly progresses to coma [24]. Fractalkine mRNA expression was downregulated in AOM-treated mice compared to time-matched vehicle controls prior to the development of neurological decline (pre), when minor neurological decline was evident (minor) and when major neurological decline was present (major) but had expression similar to controls when the mice reach coma (Fig. 1a). The expression of fractalkine was not altered in the vehicle groups over the time period studied (data not shown); therefore, all vehicle groups used in the remainder of this study were time-matched to coma AOM-treated mice. The suppression of fractalkine mRNA expression observed in AOM-treated mice correlated with a parallel reduction in fractalkine protein in cortex lysates throughout AOM-induced neurological decline (Fig. 1b). The expression of fractalkine has been reported in multiple cell types in the brain, and therefore, immunofluorescence for fractalkine was performed. This staining demonstrated that the highest expression of fractalkine was in neurons as a high degree of co-staining was found with the neuron marker NeuN (Fig. 1c). In order to determine if AOM treatment led to reduced production of neuronal fractalkine, neurons were isolated from the cortex of vehicle and AOM-treated C57Bl/6 mice using immunoprecipitation. We have previously utilized this technique and found that the neuron-enriched cell fraction had 99.7 % of the cells stain positive for CD90, which indicates that this methodology provides a relatively pure neuron cell fraction from whole brain homogenates [19]. Fractalkine mRNA expression was significantly downregulated in isolated neurons from AOM-treated mice compared to neurons isolated from vehicle-treated mice (Fig. 1d).

### CX3CR1 was downregulated in microglia following AOM-induced hepatic encephalopathy

**Fig. 2:**
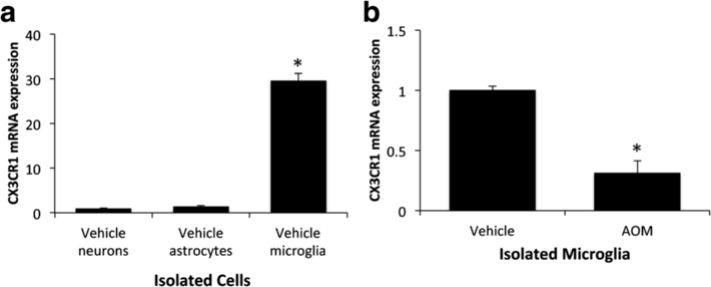
The fractalkine receptor CX3CR1 was downregulated in microglia following AOM-induced hepatic encephalopathy. **a** CX3CR1 mRNA expression in isolated neurons, astrocytes, and microglia from vehicle-treated mice with values normalized to CX3CR1 mRNA expression in vehicle neurons. **b** CX3CR1 mRNA expression in microglia isolated from the cortex of vehicle and AOM-treated mice. For mRNA analyses, *p < 0.05 compared to vehicle neurons (**a**), or microglia from vehicle-treated mice (**b**), *n* = 3 for CX3CR1 mRNA analyses

Fractalkine transduces its signal through binding CX3CR1, which has been shown in previous studies to be expressed primarily by microglia in the brain [9]. To validate these studies, the mRNA expression of CX3CR1 was measured in neurons, astrocytes, and microglia isolated from vehicle-treated mice. CX3CR1 mRNA expression was significantly higher in microglia compared to either neurons or astrocytes (Fig. 2a). As microglia are the principal cell for CX3CR1 mRNA expression in the brain, microglia were isolated from vehicle and AOM-treated mice cortices at coma using immunoaffinity isolation. This methodology had previously led to the isolation of a microglia cell fraction where 99.3 % of the cells stained positive for CD11b [19]. CX3CR1 mRNA expression was found to be significantly suppressed in isolated microglia from AOM-treated mice when compared to isolated microglia from vehicle-treated mice (Fig. 2b).

### Central infusion of soluble fractalkine improved outcomes of AOM-treated mice

**Fig. 3:**
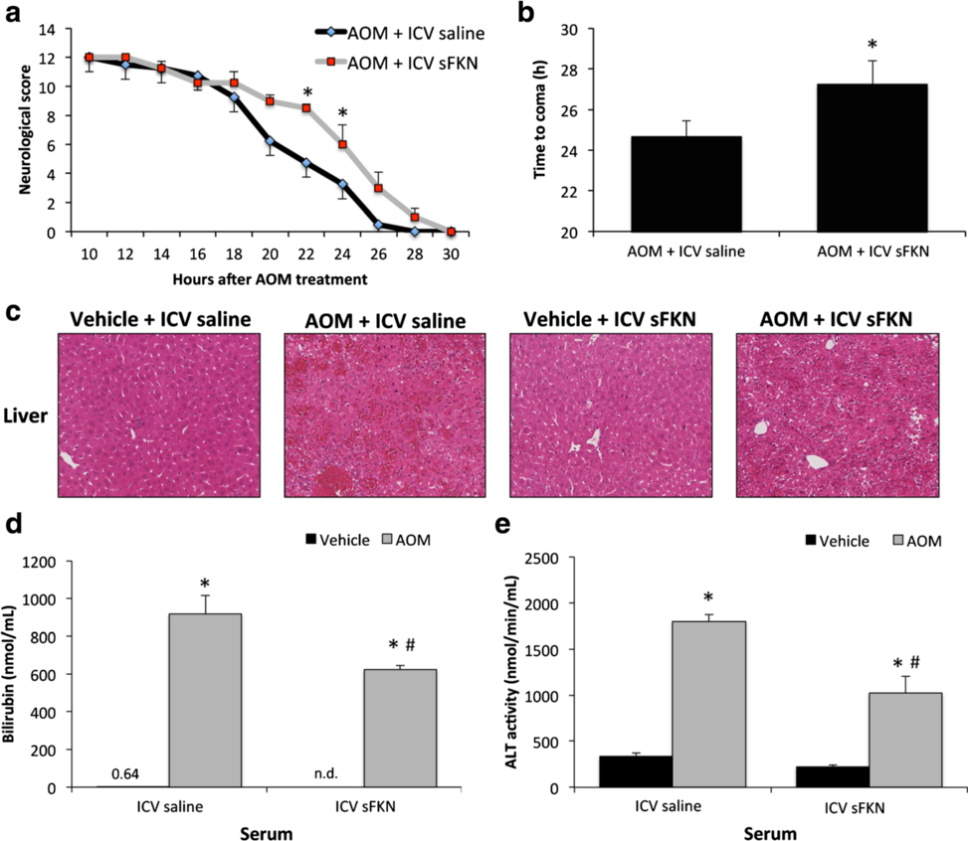
Soluble fractalkine ICV infusion improved outcomes of AOM-treated mice. **a** Neurological decline in AOM-treated mice infused ICV with either saline or soluble fractalkine (sFKN). The neurological score is a summation of assessments for five reflexes and ataxia that indicate a worse neurological state with a lower score, which is displayed in hours post AOM injection. **b** Time to coma in hours for AOM-treated mice infused with either saline or sFKN. **c** Hematoxylin and eosin staining in liver sections from vehicle or AOM-treated mice infused ICV with saline or sFKN. **d** Total serum bilirubin concentrations in vehicle or AOM-treated mice infused with saline or sFKN. **e** Serum ALT concentrations in vehicle or AOM-treated mice infused ICV with saline or sFKN. For neurological score and time to coma analyses, *p < 0.05 compared to AOM-treated mice infused with saline. For ALT and bilirubin assays, *p < 0.05 compared to vehicle-treated ICV saline mice, ^#^ *p* < 0.05 compared to AOM-treated ICV saline mice. *n* = 4 for all analyses

Due to the downregulation of both fractalkine and CX3CR1 during hepatic encephalopathy, supplementation of fractalkine could be used to induce this signaling pathway to help alleviate the pathology associated with this disease state. ICV infusion of soluble fractalkine was found to slow the neurological decline that is present during AOM-induced hepatic encephalopathy with significant differences between soluble fractalkine-infused and saline-infused AOM-treated mice particularly at 20 and 22 h following AOM injection (Fig. 3a). Furthermore, soluble fractalkine-infused mice treated with AOM took significantly longer to reach coma compared to AOM-treated mice infused with saline (Fig. 3b). To see if the neuroprotective protective effect of soluble fractalkine was due to protective effects on the liver, assessments of overall liver damage and function were performed. Hematoxylin and eosin staining in liver sections from vehicle and AOM-treated mice infused with fractalkine or saline indicates that the hepatic damage in AOM-treated mice infused with soluble fractalkine or saline is similar with significant hepatic necrosis being present in both groups (Fig. 3c). In order to better assess overall liver function, bilirubin (Fig. 3d) and ALT (Fig. 3e) concentrations were measured in serum from vehicle and AOM-treated mice infused with fractalkine or saline. The concentrations of both bilirubin and ALT in AOM-treated mice infused with saline were significantly higher than vehicle. Interestingly, bilirubin and ALT levels were significantly reduced in AOM-treated mice infused with soluble fractalkine when compared to AOM-treated mice infused with saline indicating that that ICV infusion of soluble fractalkine may improve liver function. However, both AOM-treated groups were still significantly higher than the vehicle-treated groups indicating that liver damage was still present.

### Microglia activation in AOM-treated mice was reduced by ICV infusion of soluble fractalkine

**Fig. 4:**
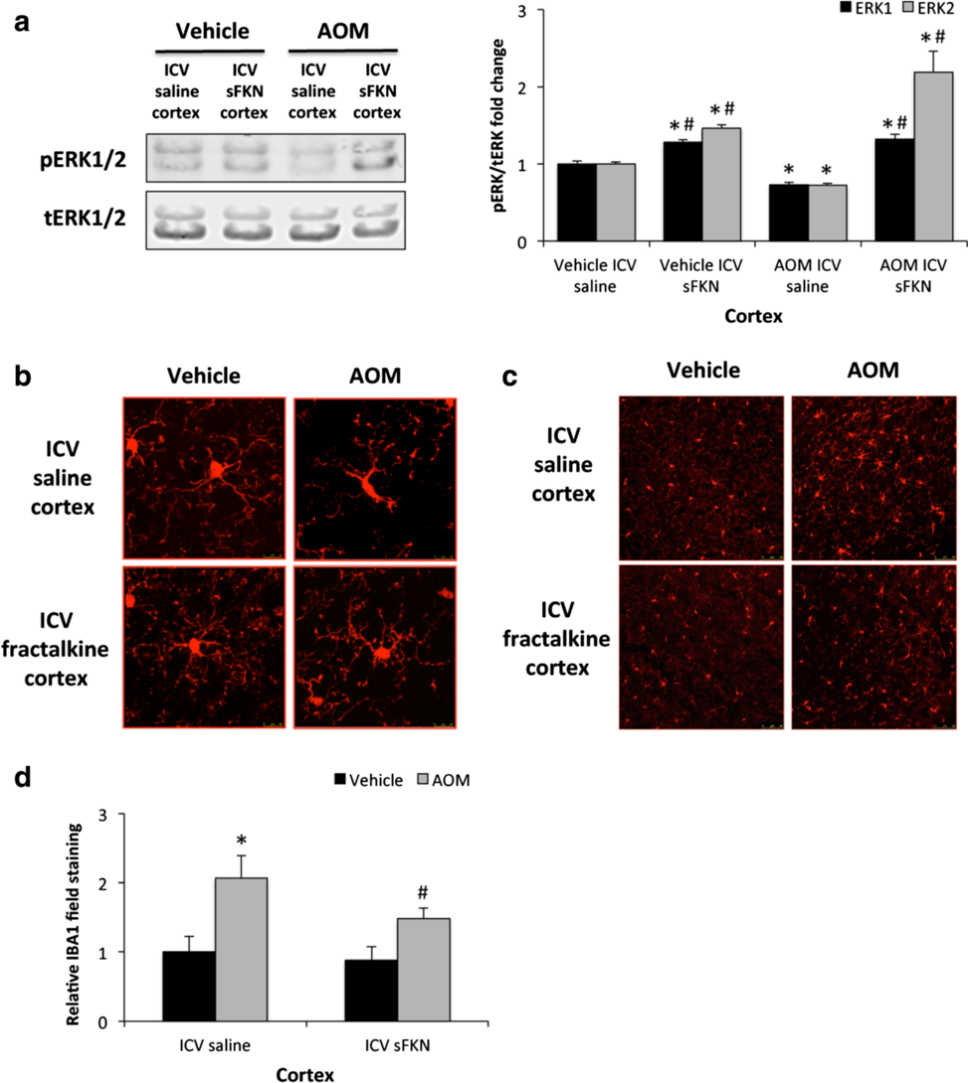
Microglia activation in AOM-treated mice was reduced by ICV infusion of soluble fractalkine. **a** Representative immunoblot images and quantification of pERK1/2 and tERK1/2 in cortex lysates from vehicle or AOM-treated mice infused ICV with saline or soluble fractalkine (sFKN). **b** Z-stack confocal images of individual microglia stained with IBA1 (*red*). Microglia when activated become more amoeboid shaped with retracted cellular processes. **c** Field staining for IBA1 (*red*) in the cortex of vehicle or AOM-treated mice infused ICV with saline or sFKN. **d** Quantification of IBA1 field staining in the cortex of vehicle or AOM-treated mice infused with saline or sFKN. For quantitative IBA1 immunofluorescence analyses, *p < 0.05 compared to vehicle-treated mice infused with saline, ^#^ *p* < 0.05 compared to AOM-treated mice infused with saline. *n* = 4 for immunoblot analyses and *n* = 10 for IBA1 field quantification analysis

Determination that soluble fractalkine infusion was generating an increase in CX3CR1-mediated signaling in the brain was accomplished by performing immunoblots for pERK1/2 and tERK1/2 as these proteins are downstream effectors of CX3CR1 signaling [25]. In the vehicle-treated mice, there was no observable difference in cortical pERK1/2 expression but pERK1/2 was reduced in AOM-treated ICV saline-infused mice compared to the vehicle-treated groups and this reduction of pERK1/2 expression was reversed by ICV soluble fractalkine infusion (Fig. 4a). As fractalkine has been reported to play a role in neuroinflammation, microglia activation was investigated. Microglia activation and proliferation has been reported in a variety of neuroinflammatory conditions to include Alzheimer’s disease and excitotoxic neuronal injury [26, 27]. One method to assess microglia activation is to determine if these cells undergo a phenotypic shift from a ramified resting state to an amoeboid active proinflammatory state. In order to examine if soluble fractalkine infusion reduces microglia activation, microglia morphology was assessed in the cortex of vehicle and AOM-treated mice that were infused with either soluble fractalkine or saline. Morphological analysis of microglia using the marker IBA-1 demonstrated that microglia in AOM-treated mice infused with saline have a larger cell body and retracted processes when compared to both vehicle-treated groups or AOM-treated mice infused with soluble fractalkine (Fig. 4b). This suggests that soluble fractalkine infusion reduced the activation state of microglia and allowed for microglia to remain in a quiescent state. In addition to phenotypic changes in microglia, neuroinflammation is also characterized by proliferation of microglia. IBA1 field staining was increased in the cortex of AOM-treated mice infused with saline, which was reduced in AOM-treated mice infused with soluble fractalkine (Fig. 4c). These data support that infusion of soluble fractalkine significantly reduced IBA1 staining in AOM-treated cortex when compared to saline-infused mice (Fig. 4d). Together, these data support that soluble fractalkine infusion reduced microglia activation and proliferation that occurs during hepatic encephalopathy.

### Elevated cytokine expression observed during hepatic encephalopathy was reduced via central fractalkine infusion

**Fig. 5:**
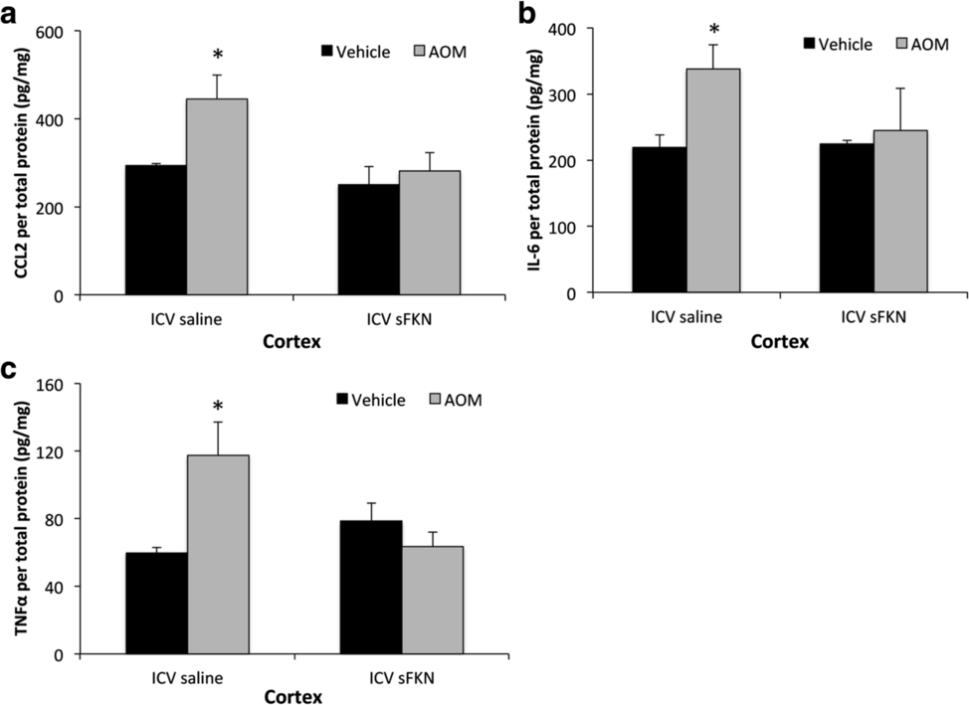
Elevated cytokine expression observed during HE can be reduced via soluble fractalkine infusion. **a** CCL2 concentrations in the cortex of vehicle or AOM-treated mice infused with saline or soluble fractalkine (sFKN) normalized for total lysate protein concentration. **b** Cortical IL-6 levels in vehicle or AOM-treated mice infused with saline or sFKN normalized for total lysate protein concentration. **c** TNFα concentrations in the cortex of vehicle or AOM-treated mice infused with saline or sFKN normalized for total lysate protein concentration. For ELISA analyses *p < 0.05 compared to vehicle-treated mice infused with saline. *n* = 3 for all ELISA analyses

Activation of microglia can lead to a variety of responses with the best characterized being the release of proinflammatory cytokines to promote neuroinflammation. CCL2 is a proinflammatory chemokine involved with the activation of microglia and has been demonstrated to play a significant role in AOM-induced hepatic encephalopathy [8]. Similar to previous studies [8], CCL2 protein expression was increased in the cortex after AOM treatment (Fig. 5a). However, pretreatment with soluble fractalkine attenuated this cortical upregulation of CCL2 expression (Fig. 5a). Downstream of chemokines are proinflammatory cytokines with both IL-6 and TNFα playing a significant role in neuroinflammation. Both IL-6 (Fig. 5b) and TNFα (Fig. 5c) were found to be elevated in the cortex of AOM-treated mice infused with saline, but expression of both cytokines was reduced following soluble fractalkine ICV infusion in AOM-treated mice. These data support the concept that soluble fractalkine ICV infusion reduces the expression of proinflammatory chemokines and cytokines during AOM-induced HE.

## Discussion

Hepatic encephalopathy is a devastating neurological complication of liver disease with few effective treatments; therefore, the need to identify novel therapeutic targets is important [28]. Taking this approach, the current study indicates the role that fractalkine signaling plays in the progression of hepatic encephalopathy through modulating microglia activation and subsequent neuroinflammation. The results from this study support that (1) fractalkine and its receptor are downregulated during AOM-induced neurological decline and (2) supplementing fractalkine levels directly in the brain reduces neurological decline, microglia activation, and the expression of proinflammatory cytokines during AOM-induced hepatic encephalopathy. These findings suggest that increasing fractalkine concentrations, which are suppressed during hepatic encephalopathy, significantly reduces inflammation and indicates that fractalkine supplementation may be a potential treatment strategy for the management of hepatic encephalopathy.

Fractalkine is a chemokine that is expressed in neurons in normal physiological conditions, and its suppression during neuroinflammatory states contributes to the elevation of proinflammatory cytokines. Indeed, fractalkine has been demonstrated to reduce both neurotoxicity and the activation of microglia in the 6-hydroxydopamine rat model of Parkinson’s disease [29]. The results from the current study indicate that during AOM-induced hepatic encephalopathy, fractalkine is downregulated in the cortex as an early event, and its expression decreases throughout the duration of this model until coma is reached. This positions fractalkine suppression as an early event in the pathogenesis of hepatic encephalopathy. One of the only other studies investigating fractalkine expression in the brain during liver pathology found that rats with partial portal vein ligation had unchanged fractalkine expression compared to control rats, though the expression of other chemokines and their receptors was significantly altered [30]. That being said, this current report is the first to investigate fractalkine signaling in overt hepatic encephalopathy due to acute liver failure and therefore, gaining understanding into fractalkine expression and signaling during other models, and patients with hepatic encephalopathy will be required to fully understand the role of this chemokine during this disease state.

In order for fractalkine to generate downstream signaling, this ligand must bind CX3CR1. As microglia are the primary source of CX3CR1, the gene expression of this receptor was assessed in isolated microglia from vehicle and AOM-treated mice. CX3CR1 mRNA was downregulated in microglia isolated from the cortices of AOM-treated mice when compared to control-treated mice. Because of the technical difficulty and low yield in isolating microglia from adult mouse brains, analysis of CX3CR1 protein was not possible in this study. Furthermore, fractalkine, via the activation of CX3CR1 leads to the downstream phosphorylation of ERK1/2. Our data support a suppression of fractalkine-induced ERK1/2 activation after AOM. Taken together, our data indicate that the fractalkine/CX3CR1 signaling axis is suppressed as an early event during AOM-induced hepatic encephalopathy that persists until coma. A similar suppression of fractalkine signaling has been reported during various other neuropathies. For example, during neuroinflammation associated with scrapie infection in hamsters, microglia activation occurs and downregulation of fractalkine and its receptor is observed [31]. Similarly, in mice with genetic knockout of CX3CR1, there is reduced expression of IL-1β and TNFα as well as reduced infarct size following focal cerebral ischemia compared to wild-type mice [32]. These effects have also been observed in the clinic, as lower plasma fractalkine concentrations have been observed in patients who have worse 6-month outcomes following stroke [33]. Similar findings regarding fractalkine were observed in the current study as ICV infusion of soluble fractalkine slowed the neurological decline and reduced the elevated concentrations CCL2, IL-1β, and TNFα that are associated with AOM-induced hepatic encephalopathy. That being said, fractalkine appears to play an immunomodulatory role that can reduce, but not completely reverse, the pathology of AOM-treated mice. AOM-induced hepatic encephalopathy is characterized by elevations of ammonia and bile acids as a result of liver injury and both of these contribute to the progression of this disorder [34]. Therefore, fractalkine supplementation may provide an even greater therapeutic benefit if used in conjunction with other therapies for the management of acute liver failure or hepatic encephalopathy.

Contrary to the data reported here, some reports in the brain, and a few investigating other inflammatory diseases, suggest that fractalkine can promote the infiltration of immune cells and generate inflammation. One of these studies demonstrates that increased fractalkine expression during experimental autoimmune encephalomyelitis, a mouse model of multiple sclerosis, promotes lymphocyte entry into the brain further exacerbating disease severity [35]. Further support of this is demonstrated in the α-synuclein-induced inflammation model of Alzheimer’s disease, where the use of CX3CR1 knockout mice has been demonstrated to reduce inflammation during this disease state compared to wild-type mice [13]. These differential effects during various neuropathies indicate that fractalkine signaling may generate different effects depending on the disease state and the pathology that contributes to the progression of the disease. Of interest is that CX3CR1 was initially thought to respond to fractalkine alone; however, more recently, CX3XR1 was found to also bind chemokine ligand 26, making the findings using CX3CR1 knockout mice difficult to interpret with respect to specific fractalkine signaling [36].

Neuroinflammation has been associated with a variety of neuropathies including multiple sclerosis, stroke, and Alzheimer’s disease [37]. In regard to hepatic encephalopathy, microglia activation has been observed during hepatic devascularization, toxic liver injury, and biliary cirrhosis [38–40]. Elevations of ammonia occur during hepatic encephalopathy and have been demonstrated to promote microglia activation in rats fed an ammonium-enriched diet indicating that ammonia may generate some of its effects on hepatic encephalopathy pathogenesis through exacerbating neuroinflammation [41]. During hepatic encephalopathy, microglia activation and subsequent neuroinflammation is thought to be primarily driven by increased concentrations of ammonia, lactate, manganese, or glutamate in the brain [42]. Currently, many of these effects are not well understood, but recent studies have begun to expand on how the initiation of neuroinflammation may occur. Neuroinflammation can be induced by chemokine signaling, such as through CCL2, which is upregulated during AOM-induced hepatic encephalopathy and chemokine receptor 2 or chemokine receptor 4 antagonist treatment improved outcomes in AOM-treated mice [8]. Similar findings were observed during bile duct ligation in mice where cerebral concentrations of CCL2 were elevated and were associated with monocyte infiltration in the brain, which was eliminated in CCL2 or chemokine receptor 2 knockout mice [40]. Of interest is that there appears to be an inverse relationship between CCL2 and fractalkine during hepatic encephalopathy. Gaining a greater understanding of the mechanism that generates these signaling events may provide better understanding of the initiators of inflammation during this disease state. This study was the first to report that supplementing fractalkine during hepatic encephalopathy suppresses CCL2 concentrations in the cortex, which indicates that the increase of CCL2 observed during AOM-induced hepatic encephalopathy is dependent upon the suppression of fractalkine. Therefore, there is support that chemokine signaling plays a significant role in the initiation of inflammation during hepatic encephalopathy, though more studies are needed to fully understand these signaling pathways and their roles in the pathogenesis of this disorder.

As AOM-induced hepatic encephalopathy is the result of acute liver injury, the finding that soluble fractalkine infusion directly into the brain conferred some protection to the liver is interesting and unexpected. Due to fractalkine being administered directly into the lateral ventricle of the brain and at a low concentration, one would logically infer that fractalkine infusion should have little effect on the liver. Recent studies have identified a variety of findings that support that this treatment could influence the liver. The first is that the blood-brain barrier becomes permeable at the later stages AOM-induced hepatic encephalopathy, which could allow small amounts of soluble fractalkine to enter the circulation at the end stages of this model [43]. That being said, fractalkine signaling during liver disease is generally thought to contribute to hepatic inflammation. In liver sections from patients with fibrosis and cirrhosis, it has been reported that hepatic stellate cells upregulate ADAMs and subsequently lead to the release of soluble fractalkine that recruits inflammatory cells to the liver and contributes to increased liver pathology [44]. This contribution to liver injury has also been shown in primary biliary cirrhosis where fractalkine triggers the infiltration of CX3CR1 expressing immune cells to the liver that promote inflammation [45]. Therefore, the protective effects of soluble fractalkine ICV infusion on the liver may be a result of improved neurological function. The connection between brain pathology and liver dysfunction is beginning to become recognized as patients with stroke and traumatic brain injury have elevations of liver enzymes following injury [46, 47]. That being said, this change in serum transaminases and liver enzymes observed during neuropathies is not well classified and gaining an understanding of brain-liver axis signaling will require more studies.

## Conclusions

The results presented suggest that following liver failure and the development of hepatic encephalopathy, fractalkine expression is reduced which contributes to the neurological decline associated with this disease. Direct cranial infusion of soluble fractalkine was found to slow neurological decline, reduce microglia activation, and decrease the concentrations of proinflammatory cytokines in the cortex. Therefore, treatments aimed at increasing fractalkine concentrations may be a potential therapeutic strategy for patients with hepatic encephalopathy.

## Abbreviations

ADAMs: A disintegrin and metalloproteinase proteins
ALT: Alanine aminotransferasase
AOM: Azoxymethane
CCL2: Chemokine ligand 2
DAPI: 4′,6-Diamidino-2-phenylindole
ERK1/2: Extracellular signal-regulated kinase 1/2
GLAST: Glutamate aspartate transporter
ICV: Intracerebroventricular
IL: Interleukin
RT-PCR: Real-time PCR
sFKN: Soluble fractalkine
TNFα: Tumor necrosis factor-α
